# Whole genome variants across 57 pig breeds enable comprehensive identification of genetic signatures that underlie breed features

**DOI:** 10.1186/s40104-020-00520-8

**Published:** 2020-12-03

**Authors:** Jingya Xu, Yuhua Fu, Yan Hu, Lilin Yin, Zhenshuang Tang, Dong Yin, Mengjin Zhu, Mei Yu, Xinyun Li, Yang Zhou, Shuhong Zhao, Xiaolei Liu

**Affiliations:** Key Laboratory of Agricultural Animal Genetics, Breeding and Reproduction, Ministry of Education & Key Laboratory of Swine Genetics and Breeding, Ministry of Agriculture & College of Animal Science and Technology, Huazhong Agricultural University, Wuhan, 430070 Hubei PR China

**Keywords:** Breed feature, CNV, GWAS, InDel, Pig, SNP

## Abstract

**Background:**

A large number of pig breeds are distributed around the world, their features and characteristics vary among breeds, and they are valuable resources. Understanding the underlying genetic mechanisms that explain across-breed variation can help breeders develop improved pig breeds.

**Results:**

In this study, we performed GWAS using a standard mixed linear model with three types of genome variants (SNP, InDel, and CNV) that were identified from public, whole-genome, sequencing data sets. We used 469 pigs of 57 breeds, and we identified and analyzed approximately 19 million SNPs, 1.8 million InDels, and 18,016 CNVs. We defined six biological phenotypes by the characteristics of breed features to identify the associated genome variants and candidate genes, which included coat color, ear shape, gradient zone, body weight, body length, and body height. A total of 37 candidate genes was identified, which included 27 that were reported previously (e.g., *PLAG1* for body weight), but the other 10 were newly detected candidate genes (e.g., *ADAMTS9* for coat color).

**Conclusion:**

Our study indicated that using GWAS across a modest number of breeds with high density genome variants provided efficient mapping of complex traits.

**Supplementary Information:**

**Supplementary information** accompanies this paper at 10.1186/s40104-020-00520-8.

## Background

With more than one billion individuals alive at any time, the domestic pig (*Sus scrofa domestica*) is one of the most important livestock animals in the world. Domestication was accomplished well before 9,000 years ago [1, 2], and a wide range of breeds were developed to meet various demands of human beings. Both domestication and artificial selection led to rapid phenotypic changes and resulted in distinct features and characteristics among breeds.

With different domestication and breeding goals, a large number of pig breeds became distributed throughout the world, and some of their characteristic features varied from one breed to another. These characteristics fall broadly into two categories, one is production features, which include growth, reproduction, carcass and meat quality, and the other consists of biological characteristics, which include body shape, appearance, and coat color. The Pietrain pig breed, which is native to Belgium, was bred as a lean pig breed and has the outstanding characteristic of high carcass lean meat percentage; the Iberian pig breed, which is native to Spain, was bred to provide high quality ham meat, but its carcass lean meat percentage is relatively low. The commercial pig breeds that include Duroc, Landrace, and Yorkshire, which are native to Europe and North America, were bred to have a large body size and exhibit excellent meat production; the Gottingen Minipig breed, which is native to Germany, was bred for biomedical research and has small body size. Coat color was always recognized as a breeding criterion, and it has been selected to satisfy breeders and customers (e.g., the coat colors of European wild boar, Duroc, Pietrain, and Yorkshire are black, brownish red, spotted, and white, respectively).

During a long period of domestication and selection, each breed formed its own production features, biological characteristics, and breed-specific genome variants features. Understanding the underlying genetic mechanism of the breed characteristic features could help breeders to develop the ideal pig breed. A genome-wide association study (GWAS) was used to detect the associations between genomic variations and phenotypic records over the past 15 years [3]. To date, many genome variants, such as single nucleotide polymorphism (SNP), insertion-deletion (InDel), and copy number variation (CNV), were associated with the traits of characteristic features. The duplication of the *KIT* gene led to the dominant white coat color [4, 5], QTLs on chromosomes 1, 5, 7, 9, and 12 were significantly associated with ear erectness and ear size [6]. InDels also showed their function by regulating gene translation or by changing the protein structure, and it is related to many traits, such as obesity [7]. Single nucleotide polymorphism (SNP) is the most commonly used type of genome variant in GWAS. However, most trait-associated SNPs reported in previous GWAS studies were found in the non-coding area and explained a small proportion of phenotypic variance [3, 8]. In contrast to SNP, insertions and deletions (InDels) are the genome variants that vary from 1 bp to 10,000 bp in length. A number of InDels are located in the coding exons and promoters of genes, and they can easily change the coding sequence of proteins that change phenotypes, especially those that cause frameshifts [9]. Copy number variation (CNV), which range from 1,000 bp to as much as a mega base, is defined as the varied copy numbers compared with the reference genome and significantly influences gene expression due to its regulation of the dosage [10, 11]. CNVs usually cause significant phenotype variation due to their influence on the levels of gene expression, and the level of gene expression increases with an increase in gene copy number [12]. CNVs play an important role in some human diseases [13], growth traits of cattle [14], and fatness in pigs [15]. Therefore, combining these three types of genome variants can improve the efficiency in identifying candidate genes for target phenotypes.

Using the publicly available, whole-genome sequencing datasets, a total of 469 pigs that composed of 57 breeds were used in this study. Approximately 19 million SNPs, 1.8 million InDels, and 18 thousand CNVs were identified. We defined six biological phenotypes by breed characteristic features, which included coat color, ear shape, gradient zone, body weight, body length, and body height. In addition, we also tried to define several production traits, which included sexual maturity, dressing percentage, and lean meat percentage. A mixed linear model (MLM) was fitted to identify the associations between genome variants and phenotypes. By using the GWAS method with public sequencing data, our results revealed that a number of genome variants and candidate genes associated with breed characteristic features have been selected during domestication and breeding.

## Methods

### Data collection

A total of 469 WGS-seq samples were downloaded from NCBI Sequence Read Archive (SRA, http://www.ncbi.nlm.nih.gov/sra/) and The European Nucleotide Archive (ENA, https://www.ebi.ac.uk/ena), 465 of them with clear *Sus scrofa* breed information (Table S1).

### Identification of short variants from WGS data

At first, the raw data was converted to fastq files using SRAToolkit (V2.8.2) [16], and the fastq files were trimmed by removing adapters and low-quality bases using Trimmomatic (v0.36) [17]. After conversion and trimming, the remaining high-quality reads were aligned against the Sscrofa11.1 reference sequence using Burrows–Wheeler Aligner 0.7.17 (BWA) [18]. The uniquely aligned reads with ≤5 mismatches were used for detection of short variants.

To obtain highly confident short variants, we employed GATK (V4.0.3.0) [19] variant calling pipelines, according to the GATK best practice online documentation. The SNPs were filtered using ‘QUAL < 30.0 || QD < 2.0 || FS > 60.0 || MQ < 40.0 || SOR > 4.0 || ReadPosRankSum < -8.0’ options, and Indels were filtered using ‘QUAL < 30.0 || QD < 2.0 || FS > 200.0 || SOR > 10.0 || ReadPosRankSum < -20.0 || MQ < 40.0 || MQRankSum < -12.5’ options. These parameters controlled not only the confidence of variation (QUAL), but also the quality of the variation in terms of read depth (QD), strand bias (FS, SOR), read bias (ReadPosRankSum), and mapping quality (MQ, MQRankSum). Finally, both SNPs and Indel VCF files were then filtered further using the vcftools with the use of the “--min-alleles 2 --max-alleles 2 --min-meanDP 5 --max-missing 0.75” options, which meant that only bi-allelic variations with depth ≥ 5 and a missing rate ≤ 25% were used for subsequent analysis. The InDels were coded as 0, 1, 2, in which 0 represented for “no insertion/deletion”, 1 represented for “insertion/deletion on one side”, 2 represented for “insertion/deletion on both sides”.

Before calling CNVs, individuals with a call rate < 95% were filtered, and a total of 432 individuals were retained for CNV calling. The CNVcaller was applied to detect the CNVRs (copy number variation regions) for all individuals with WGS data over 5X coverage according to its pipelines (https://github.com/JiangYuLab/CNVcaller) [20]. Briefly, the reference genome was divided into sliding windows that were 800 bp in length and with a step size of 400 bp. The read depth signal was calculated for each window and a GC correction was performed similar to the method used in the CNVnator [21]. To avoid the effects of individuals with different sequencing depths, the read depths were divided by the global median read depth. The CNVRs were integrated by scanning the population with aberrantly read depth, an allele frequency > 0.1, and at least three homozygous gain/loss individuals for candidate CNV windows. Finally, the CNVRs were genotyped using Gaussian mixture models for individuals with a variation percentage < 30% of the entire genome, which included 321 individuals in total. Finally, a total of 18,016 CNVs were left to be used in further analysis. According to the dosage effect theory, CNVs were coded as their copy numbers. It should be noted that if the copy number was > 6, then they were categorized into 6. The first two principal components that derived from CNV data were plotted in Fig. S1 and indicated that the samples from the same groups clustered together which meant that they represented comparatively high reliability of the CNV calling.

### Phenotype definition

Nine phenotypes were defined by the breed characteristic features in our study, which included coat color, ear shape, body height, body length, body weight, gradient zone, sexual maturity, lean meat percentage, and dressing percentage. The characteristic features of breeds were mainly from Animal Genetic Resource in China (Pigs), Wikipedia, published studies of breed genetic resources, and official websites for pig breeds. For each phenotype, breeds with no records of characteristic features were marked as missing. The nine phenotypes were defined as below (Table S2 and Supplementary Data 1):

**Coat color** was defined into five categories, which included white, spotted, multi-colored (black and white), reddish brown, and dark brown/black and coded as 1, 2, 3, 4, or 5, respectively. Breeds with various coat colors were defined as missing and coded as NA (Not Available).

**Ear shape** was defined into two categories, erect and non-erect, which were coded as 1 and 0, respectively.

**Body height** was measured from the highest point of withers to the ground, and the unit was centimeter (cm). The body height of females and males for each breed was averaged.

**Body length** was measured from the middle point of ears to the tail, and the unit was centimeter (cm). The body length of females and males for each breed was averaged.

**Body weight** was defined into three categories. The breeds with mature body weight < 100 kg were coded as 1, 100–200 kg were coded as 2, and > 200 kg were coded as 3. The body weight of females and males for each breed was averaged.

**Gradient zone** was defined as a grey belt. In many two-end-black or belted breeds, there is a grey belt composed of black skin and white hair at the junction of a white body part and a black body part. We defined this belt as the gradient zone and coded it as 1 and 0, which represented that the gradient zone existed or did not exist, respectively.

**Sexual maturity** was defined as three categories, which included premature, medium, and late, and coded as 1, 2, or 3, respectively. Breeds with sexual maturity of 1–3 months were defined as premature, 4–6 months were defined as medium, and > 6 months were defined as late maturing.

**Lean meat percentage** referred to the percentage of lean meat of a pig.

**Dressing percentage** was defined as the percentage of total weight after dressing at slaughter.

### Data quality control and imputation of SNPs and InDels

Before doing GWAS, SNPs and InDels were filtered using PLINK1.90 [22]. SNPs and InDels with call rates < 90% and minor allele frequencies (MAF) of < 0.05 were excluded. After filtering, SNPs and InDels were imputed using Beagle5.0 [23] and then SNPs and InDels were filtered with MAF < 0.05 again using PLINK1.90 [22].

Finally, a total of 469 individuals with 19,683,875 SNPs and 1,856,359 InDels were retained for subsequent data quality control based on the available individuals of each phenotype. As each phenotype had different breeds with missing values, the SNPs and InDels were then filtered with MAF < 0.05 according to the available individuals of each phenotype using PLINK 1.90 (Table S4). It should be noted that two individuals were found that may have had incorrect breed information according to the PCA results and, therefore, the phenotypes of these two individuals were recorded as missing in this study.

### Genome-wide association study

The association tests were performed by fitting a Mixed Linear Model using the following equation in the rMVP package:

$$ y= X\beta + Va+ Zu+e $$

where *y* represents the phenotype value, *β* represents the fixed effects, which includes the first three columns of principal components, *a* represents one dosage genome variant vector to be tested, and *u* represents the vector of random effects in the model that follows the distribution $u\sim N\ \left(0,G{\sigma}_{\alpha}^2\right)$, in which *G* was a genomic relationship matrix derived from all the genome-wide SNPs only [24]. *X*, *V*, and *Z* represent the incidence matrices for *β*, *a*, and *u*, respectively, and *e* is the vector of residual errors. The Bonferroni multiple test was used to correct the *P*-values, and the significant threshold was defined as 0.05/*N*, where *N* represents the number of genome variants for each independent GWAS.

### Identification of candidate genes and enrichment analysis

In this study, significant genome variants were annotated to the nearby genes within a distance of 1 Mb either downstream or upstream, and the gene annotation information was obtained from the *Sus scrofa* genome version 11.1 (www.ensembl.org/biomart/). KEGG/GO enrichment analyses were performed by TB tools toolkit [25]. Calculated *P*-values of all GO/KEGG pathways were corrected by Bonferroni correction method, considering our data was collected from public resources, we employed a loose threshold that *P*-values ≤0.05 to detect more significantly enriched GO terms/KEGG pathways for candidate genes.

## Results

### Summary of pig breeds, genome variants, and phenotypes

**Fig. 1:**
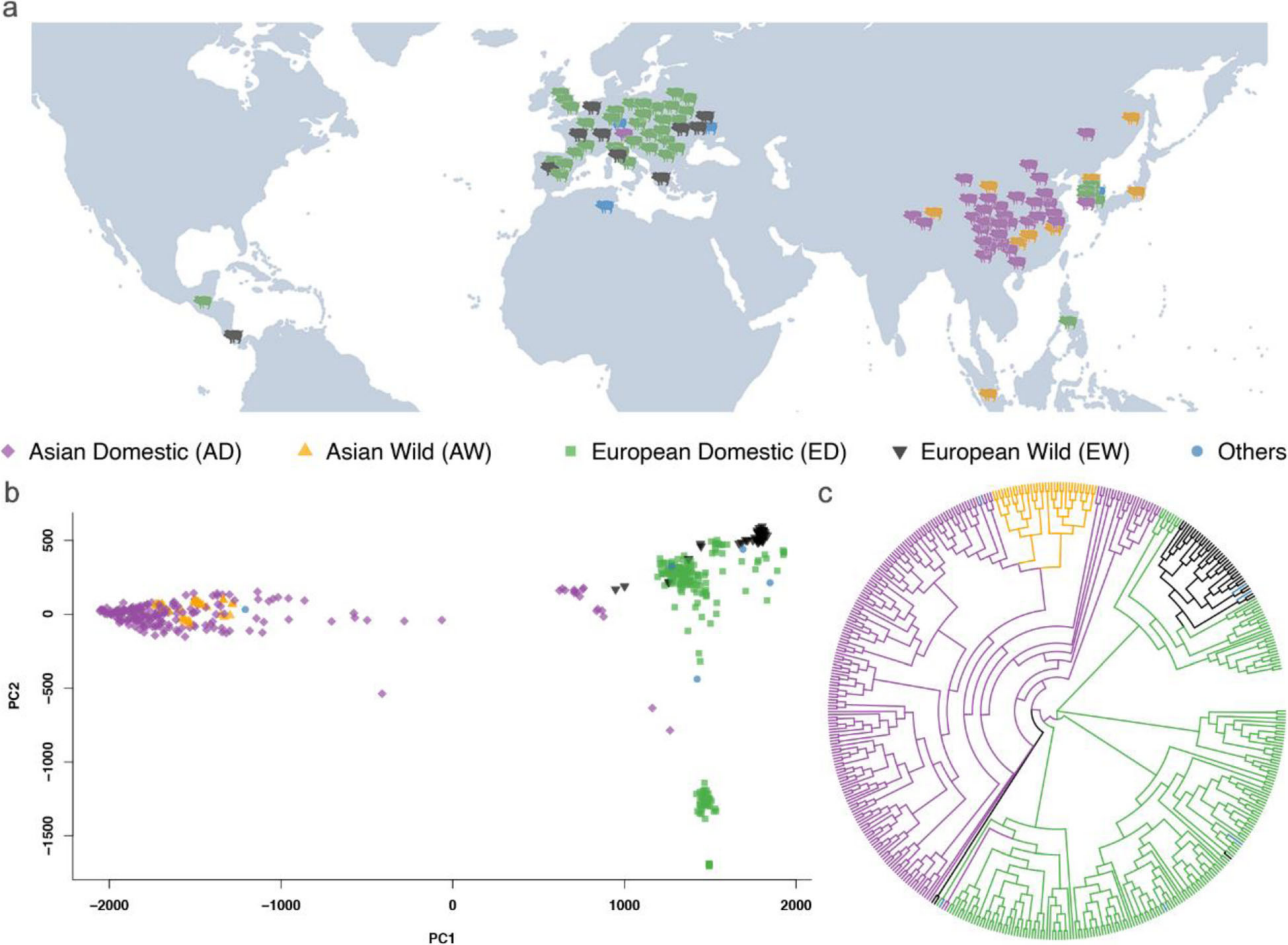
The geographical distribution, PCA plot, and phylogenetic tree of 57 pig breeds**. a** The geographical distribution of each major category of pig breeds. **b** The first two principal components derived from whole-genome SNP data. **c** The phylogenetic tree derived from the whole-genome SNP data. The pigs were clustered into five categories, which included Asian domestic (purple), Asian wild (orange), European domestic (green), European wild (black), and the breeds that do not belong to the above categories (blue)

We used 469 high-quality, whole genome sequencing datasets for an association study, which included 57 pig breeds and 9 phenotypes. The 57 pig breeds were distributed throughout the world, but they were mainly located in Asia and Europe (Fig. 1a). Both the first two principal components (PCs) and the phylogenetic tree derived from whole-genome SNP data indicated that the Asian domestic breeds (AD), Asian wild breeds (AW), European domestic breeds (ED), and European wild breeds (EW) were clustered separately [26] (Fig. 1b, c, Table 1). The phenotypes were defined by the breed features and characteristics, and the information came from Wikipedia, published genetic articles, the pigs recorded of China, which is an official record, etc. All details are described in the Methods section and Table S2.

**Table 1:** Categories of pig breeds based on geographic location and domestication

Categories	Breeds
European wild	European wild boar
European domestic	Angler Sattelschwein, Berkshire, British Saddleback, Bunte Bentheimer, Calabrese, Casertana, Chato Murciano, Cinta Senese, Creole, Duroc, Gloucester, Hampshire, Iberian, Landrace, Large Black, Leicoma, Linderodsvin, Mangalica, Middle White, Negro Iberico, Nero Siciliano, Pietrain, Retinto, Tamworth, Yorkshire, Yucatan miniature
Asian wild	Asian wild boar, Sumatra
Asian domestic	Bama Xiang, Bamei, Baoshan, Enshi Black, Erhualian, Hetao, Jeju Black, Jiangquhai, Jinhua, Laiwu, Leping Spotted, Luchuan, Meishan, Min, Neijiang, Penzhou, Rongchang, Songliao Black, Tibetan, Tongcheng, Wannan Black, Wujin, Wuzhishan, Xiang, Ya’nan
Others	African wild boar, Goettingen minipig

**Fig. 2:**
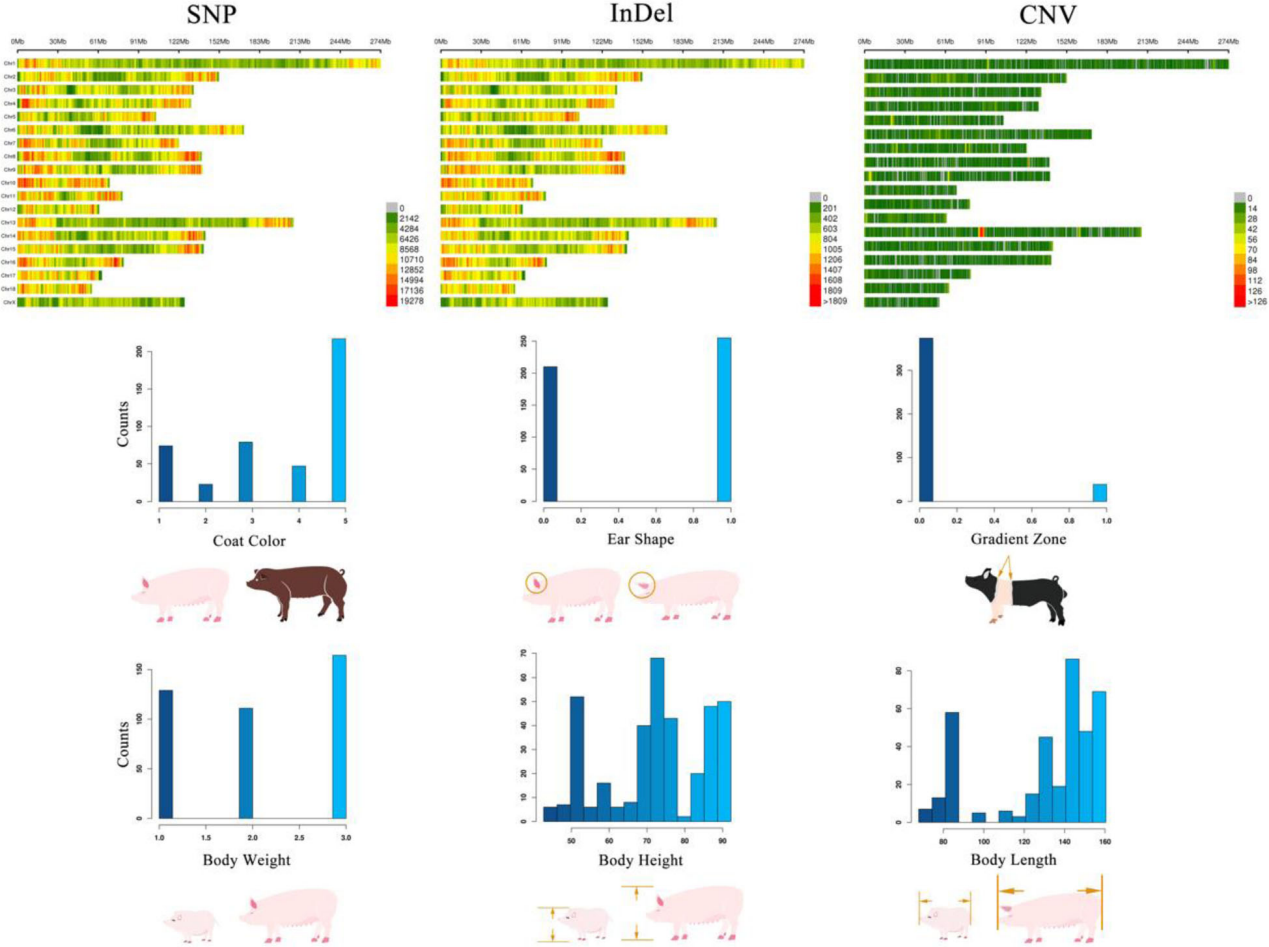
Marker density plots of three types of genome variants, phenotypic distribution plots, and schematic diagrams for six phenotypes of pig biological characteristics. The top row displays the marker density plots of SNP, InDel, and CNV, and the window size for counting genome variants was set to 1 Mb. Legends on the right side display the number of markers for each window with different colors. The histograms show the phenotypic distribution where the *X* axis represents the phenotype values and the *Y* axis represents individual counts, and schematic diagrams that depict the six phenotypes

A total of 17.93 Tb of high-quality sequencing data was aligned against the reference pig genome (Sscrofa11.1) using BWA (v0.7.17) [18]. To obtain high confidence genome variants, potential duplications were filtered for each individual; the average coverage for all individuals was approximately 93.58%, and the average depth was approximately 12.68×. The marker density plots showed that SNPs, InDels, and CNVs covered the genome well. The variations in chromosomes 1–18 and X were retained for analyses in this research, which included 469 individuals with 19,683,875 SNPs, 1,856,359 InDels, and 18,016 CNVs (Table S3). Rigorous quality control of data was conducted on the genome variants data for each phenotype (see Methods section for detail). The retained sample size and number of genome variants for further statistical analysis of each phenotype are shown in Table S4. The phenotypic distribution plots of six biological phenotypes, together with the schematic diagram of phenotype definition, are shown in Fig. 2. The rest of three production phenotypes are shown in Fig. S2.

### Summary of GWAS results

In this study, association tests were conducted using an MLM in the rMVP package (https://github.com/xiaolei-lab/rMVP). The MLM incorporated the first three columns of PCs as fixed-effect terms and the genomic relationship matrix (GRM) that represents the relationship among individuals as a random-effect term. For all three types of genome variants, both PCs and GRM were derived from the whole-genome SNP data. *P*-values were corrected by the Bonferroni multiple test method, which was defined by 0.05/*N*, where *N* represents the number of genome variants for each independent GWAS.

**Fig. 3:**
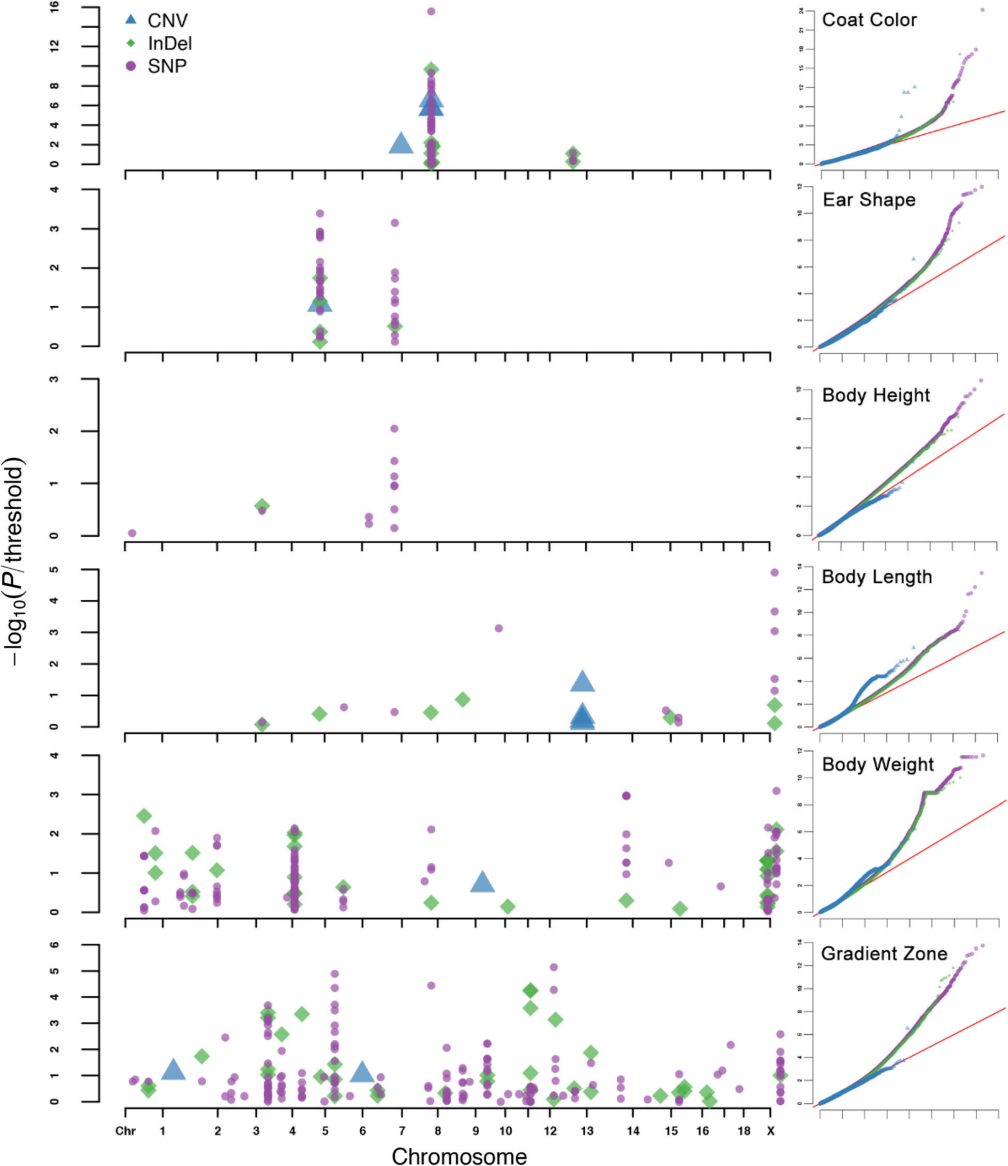
Manhattan plots and Quantile-Quantile (QQ) plots for six phenotypes of pig biological characteristics. There were three types of genome variants plotted in Manhattan plots, in which blue triangles represent CNVs, green diamonds represent InDels, and purple circles represent SNPs. Only the genome variants with genome-wide significant *P*-values were plotted in Manhattan plots, and the *P*-values were corrected by the Bonferroni cutoff, which was defined by 0.05/*N*, where *N* represents the number of genome variants for each independent GWAS. For QQ plots, the *X*-axis represents the expected −log_10_(*P*), and the y-axis represents the observed −log_10_(*P*)

The corrected *P*-values of three genome variants types were plotted simultaneously in a single Manhattan plot for each phenotype (Fig. 3). Manhattan plots for all the phenotypes are shown in Fig. S3–S11, and all the lambda values of GWASs are shown in Table S5. The estimated variance components are shown in Table S6 [27]. A total of 724 SNPs, 111 InDels, and 12 CNVs were identified to be significantly associated with six biological phenotypes (Supplementary Data 2). The gene enrichment analysis was carried out to further identify the candidate genes that were located in the candidate regions, which were defined as the genomic region that was located within 1 Mb upstream or downstream of significant genome variants (Table 2, Supplementary Data 3–11).

**Table 2:** Summary of the identified candidate regions and important genes of six biological phenotypes. For each phenotype, the candidate genes shown in bold were newly identified in this study while the un-bolded were reported previously

Phenotype	Chr	Candidate region	Type	Candidate genes
Coat color	8	40,162,068-43,262,938	SNP	*KIT, PDGFRA, KDR*
	8	46,403,615-48,403,616	SNP	***RAPGEF2***
	13	44,164,643-46,177,438	SNP	***ADAMTS9, ATXN7***
	8	40,156,302-42,630,783	InDel	*KIT, PDGFRA, KDR*
	8	45,117,702-49,266,658	InDel	***RAPGEF2,*** *PDGFC*
	13	44,164,516-46,164,570	InDel	***ADAMTS9, ATXN7***
	7	56,026,800-58,031,600	CNV	***CSPG4, PEAK1***
	8	40,222,800-42,784,000	CNV	*KIT, PDGFRA, KDR*
Ear shape	5	29,390,982-31,646,769	SNP	*MSRB3, HMGA2, WIF1, LEMD3,* ***GRIP1***
	7	29,294,596-31,559,390	SNP	*PPARD,* ***TEAD3, TULP1, BAK1***
	5	29,444,163-31,682,975	InDel	*MSRB3, HMGA2, WIF1, LEMD3,* ***GRIP1***
	7	29,303,723-31,303,724	InDel	*PPARD,* ***TEAD3, TULP1, BAK1***
	5	28,826,400-30,866,000	CNV	*MSRB3, HMGA2, WIF1, LEMD3,* ***GRIP1***
Body height	7	29,294,596-31,458,775	SNP	*PPARD, SCUBE3, HMGA1,* ***TEAD3, TULP1****, GRM4, NUDT3, RPS10, PACSIN1, SPDEF*
Body length	3	93,189,763-95,189,764	SNP	*PRKCE*
	7	29,332,971-31,332,972	SNP	*SCUBE3, HMGA1, GRM4, NUDT3, RPS10, PACSIN1, SPDEF*
	3	93,166,180-95,166,181	InDel	*PRKCE*
Body weight	1	264,450,139-266,536,416	SNP	*NR6A1*
	2	71,760,896-74,331,103	SNP	***ALKBH7***
	4	74,356,597-76,411,729	SNP	*PLAG1*
	8	11,996,794-13,996,795	SNP	*LCORL*
	17	16,190,245-18,190,246	SNP	*PLCB4*
	X	89,361,633-91,410,843	SNP	*ACSL4*
	1	264,516,132-266,534,178	InDel	*NR6A1*
	2	71,953,098-73,953,099	InDel	***ALKBH7***
	4	74,366,801-76,390,440	InDel	*PLAG1*
	15	108,303,388-110,303,389	InDel	*PARD3B*
	X	89,368,172-91,402,512	InDel	*ACSL4*
Gradient zone	5	92,898,692-94,907,622	SNP	*KITLG*
	8	40,286,000-42,286,001	SNP	*KIT, PDGFRA, KDR*
	11	49,086,896-51,103,489	SNP	*EDNRB*
	11	61,719,059-63,719,060	SNP	*DCT*
	5	92,904,398-94,906,740	InDel	*KITLG*
	11	49,076,945-51,087,682	InDel	*EDNRB*
	13	49,358,504-51,358,505	InDel	*MITF*

To track the excellent genetic resources of candidate regions, individuals of modern commercial breeds of Yorkshire, Landrace, Duroc, Pietrain, and Hampshire were separated from European domestic breeds and recognized as the fifth category, which we named commercial lines.

### Coat color

In this study, coat color was divided into five categories by the shades of black, which included white, spotted, multi-colored (black and white), reddish brown, and dark brown/black (for details, see Methods section). A total of 53 SNPs, 10 InDels, and four CNVs were associated with coat color after Bonferroni multiple corrections (Fig. 3, Table 2, Supplementary Data 2, Fig. S3).

All three types of genome variants near the *KIT* gene on chromosome 8 were significantly associated with coat color, in which a significant CNV ranges from 41,310,801 bp to 41,774,400 bp on chromosome 8 covers *KIT* gene. Three breeds of European domestic pigs, which included Leicoma, Middle White, and Calabrese, shared the same SNP/InDel genotype of the most significant signal with Yorkshire and Landrace in the commercial line. Interestingly, only Leicoma and Middle White breeds shared the same CNV genotype with Yorkshire and Landrace, and the coat color of Calabrese was black. There are normally two copies of this CNV in Calabrese, but the number of copies in Leicoma, Middle White, Yorkshire, and Landrace breeds were duplicated (> 3 copies) (Fig. S12). This suggests that the CNV may be causal for coat color instead of SNP and InDel, and this has also been proved in previous studies [4].

### Ear shape

Ear shape was classified as erect or non-erect. Two candidate regions on chromosome 5 and 7, which included 35 SNPs, five InDels, and one CNV, were significantly associated with ear shape (Fig. 3, Table 2, Supplementary Data 2, Fig. S4).

For the significant CNV that was located in the candidate region on chromosome 5, all Asian wild breeds, European wild breeds, and commercial lines of Yorkshire, Duroc, Pietrain, and Hampshire were normal (two copies), but most Asian domestic breeds and European domestic breeds were duplicated (> 3 copies). In particular, all individuals of Bamei and Min breeds had more than five copies, and their ear sizes were large, which indicated that more copies may result in larger, non-erect ears (Fig. S13).

### Body height/body length/body weight

Body size is composed of body height, body length, and body weight. Body height is defined as the mean value of female records and male records for each breed, and the same is true for body length. Mature body weights were divided into three categories, which included small (< 100 kg), medium (100–200 kg), and large (> 200 kg). A total of 11 SNPs and one InDel were associated with body height (Fig. 3, Supplementary Data 2, Fig. S5), 12 SNPs, seven InDels, and four CNVs were associated with body length (Fig. 3, Supplementary Data 2, Fig. S6), and a total of 30 candidate regions, which contained 452 significant SNPs, 53 InDels, and one CNV were associated with body weight (Fig. 3, Supplementary Data 2, Fig. S7).

The haplotypes of body size-related candidate regions in commercial lines were mainly the same, and the genotypes came from both European breeds and Asian breeds (Fig. S14–S16). The candidate region that was associated with both body height and body length on chromosome 3, the body length-related candidate region on chromosome X, and the body weight-related candidate region on chromosome 4 originated from European wild breeds. Also, the candidate regions for body weight on chromosomes 1 and X of commercial lines shared the same haplotype with Asian wild breeds. The haplotype plots for candidate regions of body size suggested that domestication in Europe and Asia was relatively independent. However, the large body size of commercial lines was formed by the genetic resources from both European breeds and Asian breeds.

### Gradient zone

Gradient zone is the grey “belt” between the black and white blocks in pigs. This phenotype was divided into two categories designated one or zero, which indicated that the gradient zone existed or did not exist, respectively. Several candidate regions that contained 161 SNPs, 35 InDels, and two CNVs were detected (Fig. 3, Supplementary Data 2, Fig. S8, Fig. S17).

## Results for the production traits

In addition to the six biological traits, we also tried to define three production traits that included sexual maturity, lean meat percentage, and dressing percentage. Details of the definition can be found in the Methods section. We found a total of seven previously reported candidate genes and no new candidate genes were identified in this study (Table S7, Supplementary Data 2, Fig. S9–S11).

### Functional enrichment

The Kyoto Encyclopedia of Genes and Genomes (KEGG) pathway and Gene Ontology (GO) enrichment analyses were performed on genes located in the candidate regions. The bubble plots of the top 10 KEGG pathways and GO terms enriched for each phenotype were shown in Fig. S18-S26.

### Coat color

**Fig. 4:**
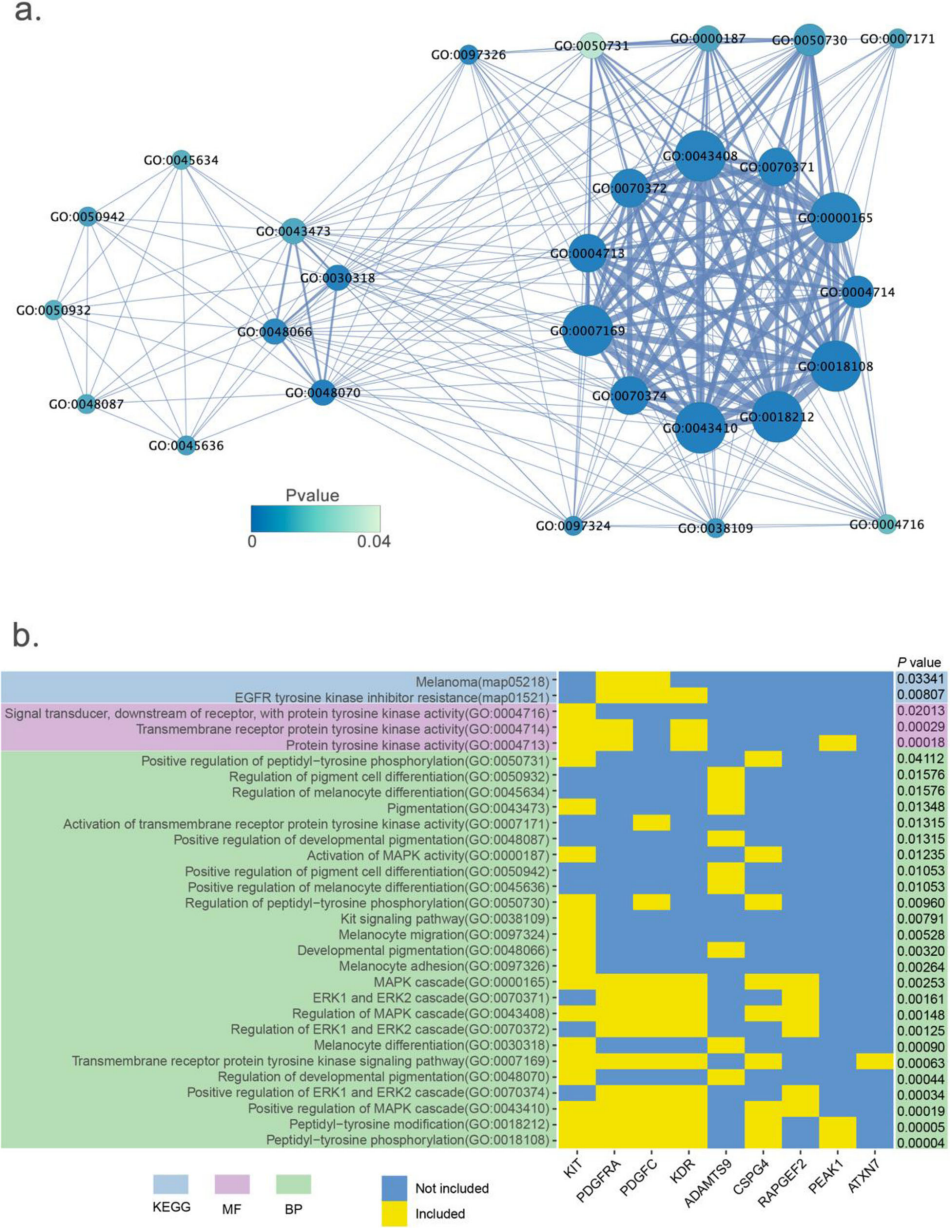
Coat color-related significant GO terms and KEGG pathways with candidate genes. **a** Significant GO terms for coat color. One overlapping gene is described by a line between each pair of two terms, where the more overlapping genes there are, the wider the lines are. The *P*-value of each term was represented by the color of each circle, and the legend showed the levels of *P*-values. **b** Heatmap of candidate genes in the coat color-related significant GO terms and KEGG pathways. The candidate genes that were identified in the coat color-related GO terms and KEGG pathways are shown at the bottom of the heatmap. If the gene was included in any GO term or pathway, it was plotted in yellow; otherwise, it was plotted in blue

A total of 68 genes located in eight candidate regions were identified as candidate genes for coat color (Supplementary Data 3). In the enrichment analysis, all candidate genes were involved in 150 KEGG pathways, and 23 of them were significant, in which some were directly related to coat color [e.g., EGFR tyrosine kinase inhibitor resistance (*P* = 8.06 × 10^−3^) and Melanoma (*P* = 3.34 × 10^−2^)]. In total, 416 significant GO terms were identified and some were pigment-related and tryosine-related, such as peptidyl-tyrosine phosphorylation (*P* = 4.28 × 10^−5^), regulation of developmental pigmentation (*P* = 4.41 × 10^−4^), pigment cell differentiation (*P* = 1.38 × 10^−3^), and melanocyte migration (*P* = 5.28 × 10^−3^) (Supplementary Data 3, Fig. S18). Within these related terms, the KIT proto-oncogene receptor tyrosine kinase (*KIT*), platelet derived growth factor receptor alpha (*PDGFRA*), kinase insert domain receptor (*KDR*), and platelet derived growth factor C (*PDGFC*) genes were reported previously as candidate genes for coat color in pigs. In addition, the Rap guanine nucleotide exchange factor 2 (*RAPGEF2*), ADAM metallopeptidase with thrombospondin type 1 motif 9 (*ADAMTS9*), Ataxin 7 (*ATXN7*), chondroitin sulfate proteoglycan 4 (*CSPG4*), and pseudopodium enriched atypical kinase 1 (*PEAK1*) were new candidate genes [28] (Fig. 4, Table 2).

### Ear shape

A total of 65 genes was detected in the candidate regions for ear shape (Supplementary Data 4). Enrichment analysis showed that some GO terms were related to ear shape, such as post-embryonic animal organ morphogenesis (*P* = 2.92 × 10^−2^) and post-embryonic animal morphogenesis (*P* = 4.35 × 10^−2^) (Supplementary Data 4, Fig. S19). However, genes within these terms, which included TEA domain transcription factor 3 (*TEAD3*), tubby like protein 1 (*TULP1*), glutamate receptor interacting protein 1 (*GRIP1*), and BCL2 antagonist/killer 1 (*BAK1*) have not been associated with ear shape before, although *GRIP1* and *TULP1* were associated with ear size in pigs. In addition, several previously reported ear-related genes were observed in these candidate regions, such as methionine sulfoxide reductase B3 (*MSRB3*), high mobility group AT-hook 2 (*HMGA2*), WNT inhibitory factor 1 (*WIF1*), LEM domain containing 3 (*LEMD3*), and peroxisome proliferator activated receptor delta (*PPARD*) (Table 2).

### Body height

In total, 99 candidate genes in candidate regions were involved in the enrichment analysis. Twelve KEGG pathways were significant from 199 KEGG pathways in total (Supplementary Data 5). There were 475 significant GO terms, of which some were skeletal-related, such as positive regulation of skeletal muscle tissue regeneration (*P* = 2.21 × 10^−2^), skeletal muscle cell differentiation (*P* = 4.42 × 10^−2^), and skeletal muscle satellite cell proliferation (*P* = 4.38 × 10^−2^) (Supplementary Data 5, Fig. S20). A KEGG pathway named Hippo signaling pathway - multiple species may be related to body height, in which the TEA domain transcription factor 3 (*TEAD3*) and tubby like protein 1 (*TULP1*) were enriched. The peroxisome proliferator activated receptor delta (*PPARD*) gene was involved in all three GO terms and was recognized as a candidate gene. In addition, the high mobility group AT-hook 1 (*HMGA1*), signal peptide, CUB domain and EGF like domain containing 3 (*SCUBE3*), glutamate metabotropic receptor 4 (*GRM4*), nudix hydrolase 3 (*NUDT3*), ribosomal protein S10 (*RPS10*), protein kinase C and casein kinase substrate in neurons 1 (*PACSIN1*), and SAM pointed domain-containing Ets transcription factor (*SPDEF*) genes were also identified as candidate genes, as reported previously (Table 2).

### Body length

A total of 223 genes in candidate regions were used to perform the enrichment analysis (Supplementary Data 6). The results revealed that 17 of 245 KEGG pathways were significant, and 458 GO terms were also significant, in which some were skeletal-related [e.g., positive regulation of skeletal muscle tissue regeneration (*P* = 4.02 × 10^−2^)] (Supplementary Data 6, Fig. S21). There were 61 overlapping genes between body length results and body height results. In these candidate regions, protein kinase C epsilon (*PRKCE*), high mobility group AT-hook 1 (*HMGA1*), and signal peptide, CUB domain and EGF like domain containing 3 (*SCUBE3*), glutamate metabotropic receptor 4 (*GRM4*), nudix hydrolase 3 (*NUDT3*), ribosomal protein S10 (*RPS10*), protein kinase C and casein kinase substrate in neurons 1 (*PACSIN1*), and SAM pointed domain-containing Ets transcription factor (*SPDEF*) genes were identified that may affect body length (Table 2).

### Body weight

We detected 366 genes in the candidate regions for body weight. Enrichment analysis showed that 31 of 269 KEGG pathways were significant, and 446 GO terms were also identified as significant (Supplementary Data 7, Fig. S22). Among the significant pathways, some were fat-related and may affect body weight, such as the PPAR signaling pathway (*P* = 1.61 × 10^−8^), Fat digestion and absorption (*P* = 3.38 × 10^−5^), regulation of lipid storage (*P* = 1.54 × 10^−2^), and positive regulation of lipid storage (*P* = 3.84 × 10^−2^) (Supplementary Data 7). According to the enrichment analysis results, the nuclear receptor subfamily 6 group A member 1 (*NR6A1*), alkB homolog 7 (*ALKBH7*), PLAG1 zinc finger (*PLAG1*), ligand dependent nuclear receptor corepressor like (*LCORL*), phospholipase C beta 4 (*PLCB4*), acyl-CoA synthetase long chain family member 4 (*ACSL4*), and par-3 family cell polarity regulator beta (*PARD3B*) were candidate genes for body weight (Table 2).

### Gradient zone

A total of 1,036 genes were detected in candidate regions for the gradient zone from enrichment analysis. Seventy one of 341 KEGG pathways were significant, and 686 GO terms were significant (Supplementary Data 8, Fig. S23). Some significant pigment-related terms may relate to the gradient zone, such as melanoma (*P* = 1.21 × 10^−4^), melanogenesis (*P* = 1.87 × 10^−3^), melanocyte differentiation (*P* = 1.03 × 10^−2^), and pigment cell differentiation (*P* = 2.19 × 10^−2^) (Supplementary Data 8). Among the genes enriched in these terms, the KIT proto-oncogene receptor tyrosine kinase (*KIT*), platelet derived growth factor receptor alpha (*PDGFRA*), kinase insert domain receptor (*KDR*), endothelin receptor type B (*EDNRB*), dopachrome tautomerase (*DCT*), microphthalmia-associated transcription factor-like (*MITF*), and kit ligand precursor (*KITLG*) were candidate genes, and all of them were associated with coat color or pigmentation.

### Enrichment results for production traits

Enrichment analyses were performed on the results of production traits (Supplementary Data 9–11). Seven previously reported candidate genes were found, such as protein kinase D1 (*PRKD1*) for sexual maturity [29].

## Discussion

In our research, we used the GWAS approach with a wide range of pig breeds to reveal the candidate genes that underlie breed characteristic features. A total of 469 pigs from 57 pig breeds with abundant genetic diversity and phenotypic diversity, which are distributed mainly in Europe and Asia, were used in this study. Nine phenotypes were defined for each breed based either on biological characteristics or production performance obtained by the information from published articles, Wikipedia, the pig breeds recorded for China, etc. (Table S2). To our knowledge, this is the first genotype-phenotype association study that used high quality whole genome variants, which included SNPs, InDels, and CNVs, in a wide range of pig breeds.

There are mainly two limitations in this study. First, with the breeding process, the phenotypic values of some breeds may be different from the latest records, which could create bias, especially for the production traits. Second, in the association test results of some traits, e.g. coat color and ear shape, more than two types of genome variants hit the same genome region and high Pearson correlation were detected among them. The regions that included more than two types of genome variants may lead to false positives because the SNPs, InDels, or the duplicated sequences may affect mapping accuracy; however, this is an inevitable general problem that lacks an appropriate solution. Furthermore, while using breed means as phenotype makes no variation in a single breed, however, this can make it possible for detecting important genes that affect phenotypes among different breeds. In addition, there are also some other methods that can be used to identify the genetic differences among multiple breeds, such as the fixation index (Fst) [30] and EigenGWAS [31]. Besides, the genetic mechanisms of some phenotypes, e.g., coat color and ear shape, might be affected by non-additive genetic systems, such as recessive alleles and/or genetic interaction. Therefore, fitting both additive and non-additive genetic effects in the model might be more suitable for those traits.

Involving many breeds in a single GWAS research could help to identify the genome variants that were domesticated or selected. However, data on multiple breeds also presented a population stratification problem. To balance the number of false positives and false negatives, and especially for controlling the false positives, we incorporated the first three PCs as fixed effects and GRM as a random effect term in a mixed linear model to correct for the population stratification, following previous studies [32–34]. Although the confounding problem between the PCs and for GRM and the tested marker decreased the power to detect candidate genes that were associated with population structure, strong signals were still identified (e.g., the KIT gene for coat color). It should be noted that because the number of CNVs and InDels were far less than SNPs, the GRM was constructed by SNPs for the association tests of all three types of genome variants. Furthermore, high-density genome variants covered the entire genome very well and reduced the dependency of linkage disequilibrium (LD) between genome variants and causal mutations to some extent, which increased the probability of success for GWAS.

The candidate regions were defined as the genomic regions that were located within 1 Mb upstream or downstream of the significant genome variants. KEGG and GO analyses were conducted to identify candidate genes. Genes that were located in the candidate regions and fell into the phenotype-related KEGG pathways, GO terms, or previously reported to be associated with the objective phenotype were considered to be candidate genes.

### Candidate genes of biological characteristics

In this study, the phenotypes of biological characteristics included coat color, ear shape, gradient zone, body length, body height, and body weight. It was obvious that these traits varied from breed to breed and showed distinct differences. *KIT*, *PDGFRA,* and *KDR* genes are well known for their key roles in the underlying mechanisms of coat color in pigs. Among the candidate genes, *KIT* on chromosome 8 was the key gene that led to the dominant white color in pigs because of the combined effect of a gene duplication and a splice mutation [4, 35]. *KIT* also played a major role in coat color of other species, such as horses and cattle [36–38]. In our study, the *KIT* gene was found in significant InDels and CNVs, which was consistent with previous studies. The *KIT* gene was reported to affect pigs’ dominant white with approximately 450-kb duplication encompassing the whole gene [5], our results hit the same genome region and identified a 463-kb duplication that contains the entire *KIT* gene. The *PDGFRA* gene on chromosome 8 was also associated with the dominant white color in pigs [39]. The *KDR* gene is close to *KIT* and *PDGFRA*, and it was a putative candidate gene for influencing coat color in cattle [40]. *KIT*, *PDGFRA,* and *KDR* genes comprise a classical cluster of tyrosine kinase receptor genes that are related to cattle’s reddish coat color [41]. *PDGFC* and *RAPGEF2* genes were also involved in the related pathways with *KIT*, *PDGFRA,* or *KDR*. In these genes, *PDGFC* was related with the two-end, black coat color in Bama Xiang pigs [42]. Related GO terms included *CSPG4*, *PEAK1*, *ADAMTS9*, and *ATXN7* (Supplementary Data 3). Among these genes, *CSPG4* and *PEAK1* were located in the candidate region near the significant CNV, and the *RAPGEF2*, *PDGFC*, *ADAMTS9*, and *ATXN7* genes were found in the candidate regions near significant InDels.

Twenty-seven genes were involved in gradient zone-related KEGG pathways, such as melanoma and melanogenesis, which included many genes (Supplementary Data 8). Among these genes, *MITF* and *EDNRB* were responsible for the two-end black trait in Chinese pig breeds [43, 44]. These two genes were identified in the InDel results, and an InDel marker within *EDNRB* was detected. Insertion of long interspersed element-1 (L1) in intron 3 of *MITF* led to a lack of melanocytes [45]. Insertion of a retroposon-like element in intron 1 of *EDNRB* caused the piebald phenotype in mice [46]. In the related GO terms, such as melanocyte differentiation and pigment cell differentiation, *KIT*, *KITLG*, *MITF*, *EDNRB*, and *DCT* were detected, in which *KITLG* was associated with the six white points in Berkshire pigs [47], and *DCT* affected coat color in mice, which coded for diluted coat color [48]. In addition, *DCT* was associated with human pigmentation in Asian populations [49].

According to the enrichment analysis results for ear shape, several pathways and GO terms were related to ear development, which included the *TEAD3*, *TULP1*, *GRIP1* and *BAK1* genes within them (Supplementary Data 4). In addition, the *HMGA2*, *PPARD*, *MSRB3*, *WIF1*, and *LEMD3* genes were found in candidate regions and affected ear size and ear morphology in pigs; *GRIP1* was also associated with ear size in pigs [50–53]. It is known that Wnt signaling regulated embryonic cartilage development and chondrocyte maturation [54], and the *PPARD* gene, which plays a key role in the Wnt/β-catenin pathway, affected ear size in pigs [55]. In a recent study, the *PPARD* gene inhibited the development of cartilage in external ears [56]. Interestingly, previous research on the same phenotypes in dogs also suggested that *HMGA2*, *WIF1*, and *MSRB3* were candidate genes [57, 58]. CNV in the *MSRB3* gene played an important role in enlarging ear size in pigs, and it has an overlap region with the significant CNV in this study [59]. In a recent canine GWAS study, *MSRB3* was also associated with drop ears, but no CNVs were reported [34].

Body height, body length, and body weight are three component phenotypes of body size, and many candidate genes were identified more than one time. In the candidate regions, 99 genes for body height and 223 genes for body length were found, in which 61 genes overlapped. *TULP1* and *TEAD3* were enriched in related pathway as said in result part. In a related GO term, positive regulation of skeletal muscle tissue regeneration, *PPARD* was identified, which was associated with limb bone length and considered as a candidate gene for body height in pigs [60] and humans [61]. Among the 61 overlapping genes, *GRM4*, *NUDT3*, *RPS10*, *PACSIN1*, *HMGA1*, *SPDEF*, and *SCUBE3* were associated with body height and body length in pigs [60, 62]. *PRKCE*, which was identified in both SNP and InDel, was associated with conformation traits in Danish breeds of pigs [63]. (Supplementary Data 5, Supplementary Data 6).

For body weight, there were 366 genes detected in the candidate regions, and many of them were enriched in lipid-related pathways that may affect body weight, such as PPAR signaling pathway, Adipocytokine signaling pathway, Fat digestion and absorption, and Regulation of lipolysis in the adipocyte. The genes (e.g., *ACSBG2*, *ACSL4*, *C1QL3*, *TRMT2B*, *PLIN3*, *PLIN5*, *CD36*, *ACBD7*, *MAPKAP1*, *INSR*, *CYP7A1*, and *GNAI1*) were included in these pathways (Supplementary Data 7). In these genes, *ACSL4* was associated with fatty acid metabolism, backfat thickness, and fatness [64–66]. Interestingly, *ACSL4* was also considered as a candidate gene for body size in dogs [67]. In the two related GO terms, regulation of lipid storage and positive regulation of lipid storage, *ALKBH7*, *C3*, and *PLIN5* were detected. A previous study reported that the deletion of *ALKBH7* caused obesity and led to an increase in body weight in *Mus musculus* [68]. In pigs, *ALKBH7* affected lipid content because of the regulation of lysine [69], therefore, it was a candidate gene for body weight. Besides these genes that were involved in related GO terms and KEGG pathways, *PLAG1*, *PLCB4*, *PARD3B*, *LCORL*, and *NR6A1* were also considered candidate genes. *PLAG1* was strongly selected and associated with the growth and fatness-related traits of pigs [70, 71]. *LCORL* was a candidate gene for body size in many other species, which included horses and cattle [72, 73]. *PLCB4* affected porcine growth traits [74, 75]. A comparative genomic study of pigs and humans reported that *PARD3B* may be a candidate gene for obesity in humans [76].

Our results indicated that combining the three types of genome variants could help to improve the efficiency of identifying candidate genes for phenotypes of interests. In some cases, candidate genes were identified by all three types of genome variants, such as the *MSRB3* gene in the results of ear shape, meanwhile, there were also some candidate genes were identified by the results of one or two genome variant types, e.g., the *CSPG4* gene, which associated with coat color, was identified by the CNV marker only; the *PARD3B*, which is a candidate gene for body weight, was identified by the InDel marker only.

### The origin of significant genome variants alleles

To trace the origin of significant genome variants alleles, the important candidate regions, which were simultaneously detected in two or three genome variants types, were selected. For each candidate region, 20 genome variants on either side of the most significant genome variants that was marked with a red star were plotted, and different genome variants alleles were colored differently (Fig. S12-S17).

Individuals were divided into Asian domestic, Asian wild, European domestic (except the commercial lines), European wild, and Commercial Lines, and the latter was composed of Yorkshire, Landrace, Duroc, Pietrain, and Hampshire. Some candidate genes revealed distinct differences between European pig breeds and Asian pig breeds. For body weight, *PLAG1* on chromosome 4 was a strong selected gene in European domestic pigs [70, 71]; our results were consistent with this conclusion and suggested that the excellent genetic resources of commercial pig breeds due to the *PLAG1* gene may have come from European wild boars (Fig. S16). In addition, Jeju Black pigs and Songliao Black pigs shared the same genotype of *PLAG1* with European domestic breeds. These pigs were also clustered closely to European domestic breeds, which suggested they may have originated from Europe or been crossed with European breeds.

## Conclusions

In this study, we examined changes in diverse biological characteristics and production performances associated with genome variants across 57 breeds in pig. A total of 37 candidate genes was identified using the GWAS approach, which included 27 that were reported previously. The other 10 were newly detected candidate genes, which constitutes a promising resource for biologically and economically important genes for pig breeding.

## Supplementary Information

**Additional file 1: Supplementary Data 1**. Breed phenotypes.

**Additional file 2: Supplementary Data 2**. Significant GWAS results for each phenotype.

**Additional file 3: Supplementary Data 3**. Candidate regions and genes in these regions with KEGG and GO results for coat color.

**Additional file 4: Supplementary Data 4**. Candidate regions and genes in these regions with KEGG and GO results for ear shape.

**Additional file 5: Supplementary Data 5**. Candidate regions and genes in these regions with KEGG and GO results for body height.

**Additional file 6: Supplementary Data 6**. Candidate regions and genes in these regions with KEGG and GO results for body length.

**Additional file 7: Supplementary Data 7**. Candidate regions and genes in these regions with KEGG and GO results for body weight.

**Additional file 8: Supplementary Data 8**. Candidate regions and genes in these regions with KEGG and GO results for gradient zone.

**Additional file 9: Supplementary Data 9**. Candidate regions and genes in these regions with KEGG and GO results for sexual maturity.

**Additional file 10: Supplementary Data 10**. Candidate regions and genes in these regions with KEGG and GO results for lean meat percentage.

**Additional file 11: Supplementary Data 11**. Candidate regions and genes in these regions with KEGG and GO results for dressing percentage.

## Data Availability

The datasets analysed during the current study are available in the NCBI Sequence Read Archive (http://www.ncbi.nlm.nih.gov/sra/) under project PRJEB1683, PRJEB9115, PRJEB9326, PRJEB9922, PRJNA144099, PRJNA176189, PRJNA186497, PRJNA213179, PRJNA221763, PRJNA231897, PRJNA238851, PRJNA239399, PRJNA240950, PRJNA254936, PRJNA255085, PRJNA260763, PRJNA273907, PRJNA281548, PRJNA309108, PRJNA314580, PRJNA320525, PRJNA322309, PRJNA354435, PRJNA393920, PRJNA398176, PRJNA41185, and PRJNA418771.

## Abbreviations

SNP: Single nucleotide polymorphism
InDel: Insertion/Deletion
CNV: Copy number variation
GWAS: Genome-wide association study
MAF: Minor allele frequency

## References

[1] Larson G, Dobney K, Albarella U, Fang M, Matisoo-Smith E, Robins J (2005). Worldwide phylogeography of wild boar reveals multiple centers of pig domestication. Science..

[2] Bosse M, Megens HJ, Frantz LA, Madsen O, Larson G, Paudel Y (2014). Genomic analysis reveals selection for Asian genes in European pigs following human-mediated introgression. Nat Commun.

[3] Visscher PM, Wray NR, Zhang Q, Sklar P, McCarthy MI, Brown MA (2017). 10 years of GWAS discovery: biology, function, and translation. Am J Hum Genet.

[4] Johansson Moller M, Chaudhary R, Hellmen E, Hoyheim B, Chowdhary B, Andersson L (1996). Pigs with the dominant white coat color phenotype carry a duplication of the KIT gene encoding the mast/stem cell growth factor receptor. Mamm Genome.

[5] Giuffra E, Tornsten A, Marklund S, Bongcam-Rudloff E, Chardon P, Kijas JM (2002). A large duplication associated with dominant white color in pigs originated by homologous recombination between LINE elements flanking KIT. Mamm Genome.

[6] Wei WH, de Koning DJ, Penman JC, Finlayson HA, Archibald AL, Haley CS (2007). QTL modulating ear size and erectness in pigs. Anim Genet.

[7] Say YH (2017). The association of insertions/deletions (INDELs) and variable number tandem repeats (VNTRs) with obesity and its related traits and complications. J Physiol Anthropol.

[8] Hindorff LA, Sethupathy P, Junkins HA, Ramos EM, Mehta JP, Collins FS (2009). Potential etiologic and functional implications of genome-wide association loci for human diseases and traits. Proc Natl Acad Sci U S A.

[9] Mills RE, Pittard WS, Mullaney JM, Farooq U, Creasy TH, Mahurkar AA (2011). Natural genetic variation caused by small insertions and deletions in the human genome. Genome Res.

[10] Zhang F, Gu W, Hurles ME, Lupski JR (2009). Copy number variation in human health, disease, and evolution. Annu Rev Genomics Hum Genet.

[11] Redon R, Ishikawa S, Fitch KR, Feuk L, Perry GH, Andrews TD (2006). Global variation in copy number in the human genome. Nature..

[12] Stranger BE, Forrest MS, Dunning M, Ingle CE, Beazley C, Thorne N (2007). Relative impact of nucleotide and copy number variation on gene expression phenotypes. Science..

[13] Ionita-Laza I, Rogers AJ, Lange C, Raby BA, Lee C (2009). Genetic association analysis of copy-number variation (CNV) in human disease pathogenesis. Genomics..

[14] Zhou Y, Utsunomiya YT, Xu L, el HA H, Bickhart DM, Alexandre PA (2016). Genome-wide CNV analysis reveals variants associated with growth traits in Bos indicus. BMC Genomics.

[15] Fowler KE, Pong-Wong R, Bauer J, Clemente EJ, Reitter CP, Affara NA (2013). Genome wide analysis reveals single nucleotide polymorphisms associated with fatness and putative novel copy number variants in three pig breeds. BMC Genomics.

[16] Kodama Y, Shumway M, Leinonen R (2012). International Nucleotide Sequence Database C. The Sequence Read Archive: explosive growth of sequencing data. Nucleic Acids Res.

[17] Bolger AM, Lohse M, Usadel B (2014). Trimmomatic: a flexible trimmer for Illumina sequence data. Bioinformatics..

[18] Li H, Durbin R (2009). Fast and accurate short read alignment with burrows-wheeler transform. Bioinformatics..

[19] McKenna A, Hanna M, Banks E, Sivachenko A, Cibulskis K, Kernytsky A (2010). The genome analysis toolkit: a MapReduce framework for analyzing next-generation DNA sequencing data. Genome Res.

[20] Wang X, Zheng Z, Cai Y, Chen T, Li C, Fu W (2017). CNVcaller: highly efficient and widely applicable software for detecting copy number variations in large populations. Gigascience..

[21] Abyzov A, Urban AE, Snyder M, Gerstein M (2011). CNVnator: an approach to discover, genotype, and characterize typical and atypical CNVs from family and population genome sequencing. Genome Res.

[22] Purcell S, Neale B, Todd-Brown K, Thomas L, Ferreira MA, Bender D (2007). PLINK: a tool set for whole-genome association and population-based linkage analyses. Am J Hum Genet.

[23] Browning SR, Browning BL (2007). Rapid and accurate haplotype phasing and missing-data inference for whole-genome association studies by use of localized haplotype clustering. Am J Hum Genet.

[24] VanRaden PM (2008). Efficient methods to compute genomic predictions. J Dairy Sci.

[25] Chen C, Xia R, Chen H, YJB H (2018). TBtools, a Toolkit for Biologists integrating various biological data handling tools with a user-friendly interface.

[26] He Z, Zhang H, Gao S, Lercher MJ, Chen WH, Hu S (2016). Evolview v2: an online visualization and management tool for customized and annotated phylogenetic trees. Nucleic Acids Res.

[27] Burch BD, Iyer HK (1997). Exact confidence intervals for a variance ratio (or heritability) in a mixed linear model. Biometrics..

[28] Otasek D, Morris JH, Boucas J, Pico AR, Demchak B (2019). Cytoscape automation: empowering workflow-based network analysis. Genome Biol.

[29] Nonneman DJ, Schneider JF, Lents CA, Wiedmann RT, Vallet JL, Rohrer GA (2016). Genome-wide association and identification of candidate genes for age at puberty in swine. BMC Genet.

[30] Meirmans PG, Hedrick PW (2011). Assessing population structure: F (ST) and related measures. Mol Ecol Resour.

[31] Chen GB, Lee SH, Zhu ZX, Benyamin B, Robinson MR (2016). EigenGWAS: finding loci under selection through genome-wide association studies of eigenvectors in structured populations. Heredity (Edinb).

[32] Fang L, Wang Q, Hu Y, Jia Y, Chen J, Liu B (2017). Genomic analyses in cotton identify signatures of selection and loci associated with fiber quality and yield traits. Nat Genet.

[33] Zhou Z, Li M, Cheng H, Fan W, Yuan Z, Gao Q (2018). An intercross population study reveals genes associated with body size and plumage color in ducks. Nat Commun.

[34] Plassais J, Kim J, Davis BW, Karyadi DM, Hogan AN, Harris AC (2019). Whole genome sequencing of canids reveals genomic regions under selection and variants influencing morphology. Nat Commun.

[35] Marklund S, Kijas J, Rodriguez-Martinez H, Ronnstrand L, Funa K, Moller M (1998). Molecular basis for the dominant white phenotype in the domestic pig. Genome Res.

[36] Marklund S, Moller M, Sandberg K, Andersson L (1999). Close association between sequence polymorphism in the KIT gene and the roan coat color in horses. Mamm Genome.

[37] Brooks SA, Bailey E (2005). Exon skipping in the KIT gene causes a Sabino spotting pattern in horses. Mamm Genome.

[38] Fontanesi L, Tazzoli M, Russo V, Beever J (2010). Genetic heterogeneity at the bovine KIT gene in cattle breeds carrying different putative alleles at the spotting locus. Anim Genet.

[39] Johansson M, Ellegren H, Marklund L, Gustavsson U, Ringmar-Cederberg E, Andersson K (1992). The gene for dominant white color in the pig is closely linked to ALB and PDGRFRA on chromosome 8. Genomics..

[40] Meszaros G, Petautschnig E, Schwarzenbacher H, Solkner J (2015). Genomic regions influencing coat color saturation and facial markings in Fleckvieh cattle. Anim Genet.

[41] Hanna LL, Sanders JO, Riley DG, Abbey CA, Gill CA (2014). Identification of a major locus interacting with MC1R and modifying black coat color in an F (2) Nellore-Angus population. Genet Sel Evol.

[42] Yang Y, Adeola AC, Xie HB, Zhang YP (2018). Genomic and transcriptomic analyses reveal selection of genes for puberty in Bama Xiang pigs. Zool Res.

[43] Wang C, Wang HY, Zhang Y, Tang ZL, Li K, Liu B (2015). Genome-wide analysis reveals artificial selection on coat colour and reproductive traits in Chinese domestic pigs. Mol Ecol Resour.

[44] Ai H, Huang L, Ren J. Genetic diversity, linkage disequilibrium and selection signatures in chinese and Western pigs revealed by genome-wide SNP markers. PLoS One. 2013;8:e56001. 10.1371/journal.pone.0056001 PMC3567019 23409110

[45] Takeda K, Hozumi H, Nakai K, Yoshizawa M, Satoh H, Yamamoto H (2014). Insertion of long interspersed element-1 in the Mitf gene is associated with altered neurobehavior of the black-eyed white Mitf (mi-bw) mouse. Genes Cells.

[46] Yamada T, Ohtani S, Sakurai T, Tsuji T, Kunieda T, Yanagisawa M (2006). Reduced expression of the endothelin receptor type B gene in piebald mice caused by insertion of a retroposon-like element in intron 1. J Biol Chem.

[47] Okumura N, Matsumoto T, Hamasima N, Awata T (2008). Single nucleotide polymorphisms of the KIT and KITLG genes in pigs. Anim Sci J.

[48] Guyonneau L, Murisier F, Rossier A, Moulin A, Beermann F (2004). Melanocytes and pigmentation are affected in dopachrome tautomerase knockout mice. Mol Cell Biol.

[49] McEvoy B, Beleza S, Shriver MD (2006). The genetic architecture of normal variation in human pigmentation: an evolutionary perspective and model. Hum Mol Genet.

[50] Zhang Y, Liang J, Zhang L, Wang L, Liu X, Yan H, et al. Porcine methionine sulfoxide reductase B3: molecular cloning, tissue-specific expression profiles, and polymorphisms associated with ear size in *Sus scrofa*. J Anim Sci Biotechnol. 2015;6:60. 10.1186/s40104-015-0060-x PMC4696113 26719797

[51] Li PH, Xiao SJ, Wei N, Zhang ZY, Huang RH, Gu YQ (2012). Fine mapping of a QTL for ear size on porcine chromosome 5 and identification of high mobility group AT-hook 2 (HMGA2) as a positional candidate gene. Genet Sel Evol.

[52] Zhang LC, Liang J, Luo WZ, Liu X, Yan H, Zhao KB (2014). Genome-wide scan reveals LEMD3 and WIF1 on SSC5 as the candidates for porcine ear size. PLoS One.

[53] Wilkinson S, Lu ZH, Megens HJ, Archibald AL, Haley C, Jackson IJ (2013). Signatures of diversifying selection in European pig breeds. PLoS Genet.

[54] Macsai CE, Foster BK, Xian CJ (2008). Roles of Wnt signalling in bone growth, remodelling, skeletal disorders and fracture repair. J Cell Physiol.

[55] Ren J, Duan Y, Qiao R, Yao F, Zhang Z, Yang B (2011). A missense mutation in PPARD causes a major QTL effect on ear size in pigs. PLoS Genet.

[56] Zhang Z, Duan YY, Wu ZP, Zhang H, Ren J, Huang LS (2017). PPARD is an inhibitor of cartilage growth in external ears. Int J Biol Sci.

[57] Boyko AR, Quignon P, Li L, Schoenebeck JJ, Degenhardt JD, Lohmueller KE (2010). A simple genetic architecture underlies morphological variation in dogs. PLoS Biol.

[58] Vaysse A, Ratnakumar A, Derrien T, Axelsson E, Rosengren Pielberg G, Sigurdsson S (2011). Identification of genomic regions associated with phenotypic variation between dog breeds using selection mapping. PLoS Genet.

[59] Chen C, Liu C, Xiong X, Fang S, Yang H, Zhang Z, et al. Copy number variation in the MSRB3 gene enlarges porcine ear size through a mechanism involving miR-584-5p. Genet Sel Evol. 2018;50:72. 10.1186/s12711-018-0442-6 PMC6307293 30587124

[60] Zhang LC, Li N, Liu X, Liang J, Yan H, Zhao KB, et al. A genome-wide association study of limb bone length using a Large White × Minzhu intercross population. Genet Sel Evol. 2014;46:56. 10.1186/s12711-014-0056-6 PMC4219012 25366846

[61] Allen HL, Estrada K, Lettre G, Berndt SI, Weedon MN, Rivadeneira F (2010). Hundreds of variants clustered in genomic loci and biological pathways affect human height. Nature..

[62] Wang L, Zhang L, Yan H, Liu X, Li N, Liang J, et al. Genome-wide association studies identify the loci for 5 exterior traits in a Large White x Minzhu pig population. PLoS One. 2014;9:e103766. 10.1371/journal.pone.0103766 PMC4121205 25090094

[63] Le TH, Christensen OF, Nielsen B, Sahana G. Genome-wide association study for conformation traits in three Danish pig breeds. Genet Sel Evol. 2017;49:12. 10.1186/s12711-017-0289-2 PMC5259967 28118822

[64] Mercade A, Estelle J, Perez-Enciso M, Varona L, Silio L, Noguera JL (2006). Characterization of the porcine acyl-CoA synthetase long-chain 4 gene and its association with growth and meat quality traits. Anim Genet.

[65] Ma J, Gilbert H, Iannuccelli N, Duan Y, Guo B, Huang W, et al. Fine mapping of fatness QTL on porcine chromosome X and analyses of three positional candidate genes. BMC Genet. 2013;14:46. 10.1186/1471-2156-14-46 PMC3691627 23725562

[66] Cepica S, Bartenschlager H, Geldermann H (2007). Mapping of QTL on chromosome X for fat deposition, muscling and growth traits in a wild boar x Meishan F-2 family using a high-density gene map. Anim Genet.

[67] Plassais J, Rimbault M, Williams FJ, Davis BW, Schoenebeck JJ, Ostrander EA. Analysis of large versus small dogs reveals three genes on the canine X chromosome associated with body weight, muscling and back fat thickness. PLoS Genet. 2017;13:e1006661. 10.1371/journal.pgen.1006661 PMC5357063 28257443

[68] Solberg A, Robertson AB, Aronsen JM, Rognmo O, Sjaastad I, Wisloff U (2013). Deletion of mouse Alkbh7 leads to obesity. J Mol Cell Biol.

[69] Wang TJ, Feugang JM, Crenshaw MA, Regmi N, Blanton JR, Liao SFF. A systems biology approach using Transcriptomic data reveals genes and pathways in porcine skeletal muscle affected by dietary lysine. Int J Mol Sci. 2017;18(4):885. 10.3390/ijms18040885 PMC5412465 28430144

[70] Qiao R, Gao J, Zhang Z, Li L, Xie X, Fan Y (2015). Genome-wide association analyses reveal significant loci and strong candidate genes for growth and fatness traits in two pig populations. Genet Sel Evol.

[71] Rubin CJ, Megens HJ, Martinez Barrio A, Maqbool K, Sayyab S, Schwochow D (2012). Strong signatures of selection in the domestic pig genome. Proc Natl Acad Sci U S A.

[72] Metzger J, Schrimpf R, Philipp U, Distl O. Expression levels of LCORL are associated with body size in horses. PLoS One. 2013;8:e56497. 10.1371/journal.pone.0056497 PMC3572084 23418579

[73] Lindholm-Perry AK, Sexten AK, Kuehn LA, Smith TPL, King DA, Shackelford SD, et al. Association, effects and validation of polymorphisms within the NCAPG - LCORL locus located on BTA6 with feed intake, gain, meat and carcass traits in beef cattle. BMC Genetics. 2011;12:103. 10.1186/1471-2156-12-103 PMC3287254 22168586

[74] Edea Z, Hong JK, Jung JH, Kim DW, Kim YM, Kim ES (2017). Detecting selection signatures between Duroc and Duroc synthetic pig populations using high-density SNP chip. Anim Genet.

[75] Fontanesi L, Schiavo G, Galimberti G, Calo DG, Russo V (2014). A genomewide association study for average daily gain in Italian large white pigs. J Anim Sci.

[76] Kim J, Lee T, Kim TH, Lee KT, Kim H. An integrated approach of comparative genomics and heritability analysis of pig and human on obesity trait: evidence for candidate genes on human chromosome 2. BMC Genomics. 2012;13:711. 10.1186/1471-2164-13-711 PMC3562524 23253381

